# Rigor and reproducibility in polymer nanoparticle synthesis and characterization†

**DOI:** 10.1039/c9ra10091a

**Published:** 2020-01-14

**Authors:** Kenneth R. Sims, Julian P. Maceren, Alexander Ian Strand, Brian He, Clyde Overby, Danielle S. W. Benoit

**Affiliations:** Dept. of Biomedical Engineering, University of Rochester Rochester NY USA benoit@bme.rochester.edu; Translational Biomedical Science, University of Rochester School of Medicine and Dentistry Rochester NY USA; Dept. of Chemistry, University of Rochester Rochester NY USA; Dept. of Statistics, University of Rochester Rochester NY USA; Materials Science Program Rochester NY USA; Center for Oral Biology, University of Rochester Rochester NY USA; Center for Musculoskeletal Research, University of Rochester Rochester NY USA; Dept. of Chemical Engineering, University of Rochester Rochester NY USA

## Abstract

Standardized process improvement methods and tools were used to enhance the rigor and reproducibility of diblock copolymer nanoparticle (NP) synthesis and characterization. Models linking design parameters with NP characteristics boosted process control for NP synthesis, which may improve translation and commercialization of NP research.

## Abstract 2

Novel modeling and process control approaches provide useful insights to improve rigor and reproducibility in polymer nanoparticle synthesis and characterization.

## Graphical abstract

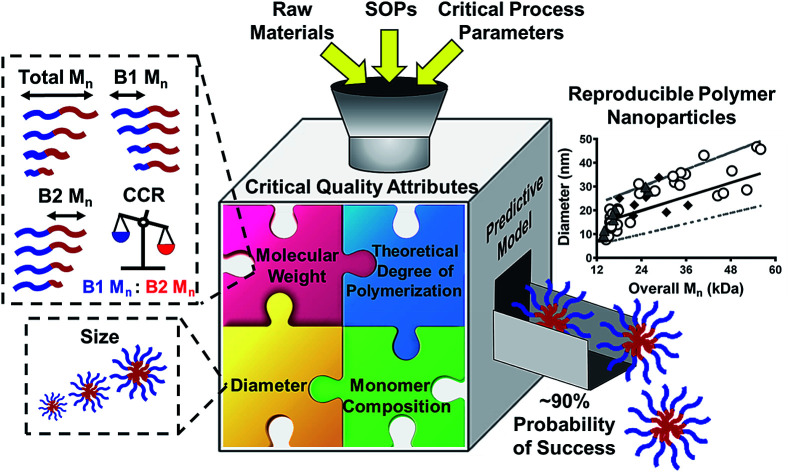

The field of nanomedicine is rapidly expanding. Nanomedicine exploits nanoparticles (NPs) to improve therapeutic targeting, control drug release, reduce off-target toxicity, and/or enhance therapeutic efficacy.^1–5^ Since the National Nanotechnology Initiative (NNI) was established in the early 2000s, the US Food and Drug Administration has approved more than 50 nanomedicines, and dozens more are in development or undergoing clinical trials.^2,3^ However, clinical translation of NP research has been rather modest considering the large NNI investment (*e.g.*, >$20 billion).^2,6–8^ The latest assessment of the NNI defined the second era of nanotechnology (*i.e.*, NNI 2.0) as a pivotal crossroads where sustained program success hinges on effective, rapid translation of nanomedicine research into products.^7^ Concurrently, as part of the National Institutes of Health (NIH) Roadmap for Medical Research, the NIH announced new guidelines (*e.g.*, NOT-OD-16-011) requiring evaluation of experimental rigor and reproducibility in research grant applications and progress reports. Together, the NNI assessment and new NIH guidelines highlighted a need for standardization in NP research.^9,10^

NNI 2.0 success requires standard NP production processes.^7,9,10^ In particular, strategies to translate nanomedicines from lab scale to full scale manufacturing must minimize heterogeneity due to process variation.^7,9^ The application of Six Sigma principles and statistical methods is one way to ensure process uniformity.^11,12^ Moreover, robust characterization studies correlating product critical quality attributes (CQAs), such as number average molecular weight (*M*_n_) and size (*e.g.*, diameter), across a wide design space are imperative for clinical translation and commercialization of polymer NPs. The current lack of CQA studies likely stems from narrow experimental designs using small sample sizes and arbitrary, application-specific test conditions. Instead, reports of rigorously designed and carefully executed experiments evaluating multiple samples across a variety of conditions that yield reproducible results would greatly benefit translational endeavors. A recent article aptly delineated a ‘minimum information standard’ to improve the fundamental understanding of nanomaterials interactions with biological systems (*i.e.*, bio-nano interactions).^13^ This standard introduced a checklist approach to define what information should be collected and published for new nanomaterials intended for biological applications with specific emphasis on material and biological characterizations and experimental protocol details.^13^ This guidance will undoubtedly improve translational outcomes in the nanomedicine field if universally adopted. Additionally, more study results should be made publicly available considering recent reports suggest as little as 12% of nanotechnology research data is currently published or accessible *via* electronic databases.^14^ A potential remedy to these challenges is the application of Lean principles to improve study design and process execution by reducing waste and improving the value of bench scale NP studies.^11^ By improving the robust collection, storage, and dissemination of standardized, rigorous and reproducible nanotechnology data, collective learning across the field will occur more rapidly and likely expedite clinical translation and commercialization of nanoscale research into FDA-approved products. Thus, this study exploits strategies to improve rigor and reproducibility in NP research and development *via* the application of Lean and Six Sigma principles and practices.

Specifically, the feasibility of implementing standardized continuous improvement methods to enhance the rigor and reproducibility of polymer NP research was demonstrated. As an archetypical approach, an established polymer NP platform (Fig. S1†) capable of delivering a wide variety of drug cargos to mammalian and bacterial cells was used.^15–25^ Given the versatility of this NP platform and how CQAs (*e.g.*, polymer *M*_n_; NP core design and diameter) influence its functional efficacy,^15,16,20–22^ a rigorous design model and a reproducible production process is essential for clinical translation. By applying Lean Six Sigma (LSS) and process monitoring principles to polymer synthesis and characterization methods, reproducibility was achieved across personnel, analytical equipment, and reaction scale. LSS principles and practices were chosen over other processes, such as workflow mapping and Shewart cycles, due to the reliance of LSS on efficiency between process steps and value added data collection (*e.g.*, Lean) as well as data-centered techniques and analysis (*e.g.*, Six Sigma).^11,12,26^ On both fronts, LSS pairs well with analytical research activities involving complex procedures to synthesize, purify, characterize, and model drug delivery vehicles in preclinical settings. Implementation of these practices may improve polymer NP translational success. Recommendations are also offered on how to best apply these practices in research laboratories by building on previously published advice regarding the use of Lean and Six Sigma techniques in clinical and translational research.^11^

Diblock copolymer NPs were synthesized using a two-step reversible-addition fragmentation chain transfer (RAFT) polymerization process, as described previously (see ESI†).^19–21^ The controlled radical-mediated reaction rates together with flexibility towards a variety of reaction conditions^27–29^ makes RAFT particularly amenable to process control methodologies. The diblock copolymers consisted of dimethylaminoethyl methacrylate (DMAEMA) as the hydrophilic first block (*i.e.*, Block 1) and a combination of DMAEMA, butyl methacrylate (BMA), and propyl acrylic acid (PAA) as the hydrophobic second block (*i.e.*, Block 2). The syntheses were completed per standard operating procedures (SOPs) that precisely defined critical process parameters (CPPs), such as reactant masses, measurement tolerances, reaction times, and temperatures. Indeed, the use of clear, concise, and comprehensive SOPs in research laboratories has received increased attention in recent years^30,31^ and is one area where the nanotechnology research community can collaborate and improve translation of nanomedicines to the clinic by sharing standardized NP synthesis and characterization methods.^13,32^

Multiple Block 1 and Block 2 polymer syntheses were performed (Alpha results in Fig. 1A and B) to obtain pilot empirical *M*_n_ data for each block as a function of a key SOP calculation parameter: theoretical Degree of Polymerization (DP). Theoretical DP corresponds to the molar ratio of monomer and chain transfer agent used in a reaction. Thus, theoretical DP is a CPP that directly impacts Block 1 and Block 2 *M*_n_ and may aid Quality by Design (QbD) practices.^33,34^

**Fig. 1:**
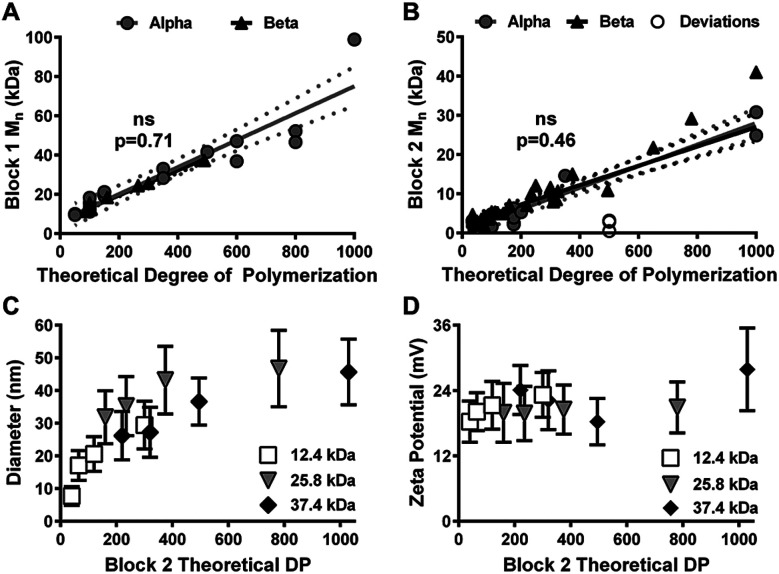
Polymer and nanoparticle (NP) characterization as a function of theoretical Degree of Polymerization (DP). Scatterplots of Block 1 (A) and Block 2 (B) number average molecular weight (*M*_n_) as a function of theoretical DP for Alpha (grey circles) and Beta (black triangles) syntheses. White circles represent polymers with process deviations. Data are shown as individual *M*_n_ values and linear regressions for each group. ns = no significant difference between the regression slopes per analysis of covariance testing. Dotted lines represent 95% confidence intervals for linear regression lines. Scatterplots of NP diameter (C) and zeta potential (D) *versus* Block 2 theoretical DP for copolymers synthesized using different Block 1s (12.4 kDa, white squares; 25.8 kDa, grey triangles; 37.4 kDa, black diamonds). Data are shown as mean ± standard deviation from *n* = 3 independent size measurements (C) and *n* = 5 independent zeta potential measurements (D).

As shown in Fig. 1, theoretical DP was used to determine the variance of CQA parameters (*e.g.*, *M*_n_, diameter, or zeta potential). Based on these results, the Alpha syntheses yielded coefficient of determination (*R*^2^) values 0.88 and 0.93 for Block 1 *M*_n_ (Fig. 1A) and Block 2 *M*_n_ (Fig. 1B), respectively, *versus* theoretical DP. Calculated slopes and intercepts were used to predict desired *M*_n_s from theoretical DP values, and Beta syntheses were carried out to test model accuracy. Beta syntheses results closely aligned with modeled expectations (*p* > 0.05, Fig. 1A and B). However, the use of this model for the Beta syntheses only improved *R*^2^ for Block 1 *M*_n_ (*e.g.*, 0.98) while *R*^2^ for Block 2 *M*_n_ decreased to 0.76. This result was due to a combination of normal cause variation associated with macro chain transfer agent efficiency and Block 2 monomer reactivity rates (data not shown) as well as special cause variation associated with gel permeation chromatography (GPC) analytical column changes and different personnel performing the syntheses (as shown in Fig. S2 and S3†).

Regardless, the pilot empirical data formed the basis of a prediction model used to reproducibly synthesize polymer NPs with specific Block 1 and Block 2 *M*_n_s. This model also served as a useful tool for continuous process monitoring and quality control; polymers that experienced major process deviations became apparent when compared to polymers synthesized according to SOPs (Fig. 1B). Similar plots evaluating NP diameter (Fig. 1C) and zeta potential (Fig. 1D) as a function of Block 2 theoretical DP were also considered. Although no differences in zeta potential were observed (Fig. 1D), NP diameter appeared to correlate non-linearly with Block 2 theoretical DP (Fig. 1C) albeit with poor fits (*R*^2^ = 0.34–0.73 using second order polynomial and logarithmic fits). Altogether, results shown in Fig. 1 revealed that desired copolymer *M*_n_ and NP diameter could be controlled by adjusting a single batch parameter: theoretical DP. Future efforts to develop and implement similar modeling methods will improve process reproducibility and significantly aid future translation of polymer NP systems.

A more robust modeling scheme was explored by leveraging the transitive relationship between polymer *M*_n_ and NP diameter given that both parameters modestly correlated with theoretical DP. Changes in NP diameter were examined as overall diblock copolymer *M*_n_ (Fig. 2A(i)), DMAEMA *M*_n_ (Fig. 2A(ii)), and monomer repeats within the hydrophobic core (Fig. 2B(i), DMAEMA; Fig. 2B(ii), BMA; Fig. 2(iii), PAA) changed across 32 polymers. Except for DMAEMA repeats (Fig. 2B(i)), each evaluation yielded somewhat linear correlations. BMA repeats and overall *M*_n_ exhibited the highest degree of linearity with *R*^2^ values of 0.78 and 0.72, agreeing with previous reports.^20,35–37^ Furthermore, PAA repeats exhibited a modest linear *R*^2^ value (0.62) while DMAEMA *M*_n_ exhibited a poor fit (*R*^2^ = 0.42). Performance evaluation for multiple process variables, such as lab personnel (*i.e.*, personnel A–D, Fig. S2†) and analytical equipment (*i.e.*, GPC columns V–Z, Fig. S3†) was also completed based on these data. Stratification of process performance data in Fig. S2 and S3† can help identify potential sources of variability or key process changes over time and thus provide vital insights for continuous process improvement. However, this information must be aptly documented for each polymer batch to make it useful for retrospective data analyses.

**Fig. 2:**
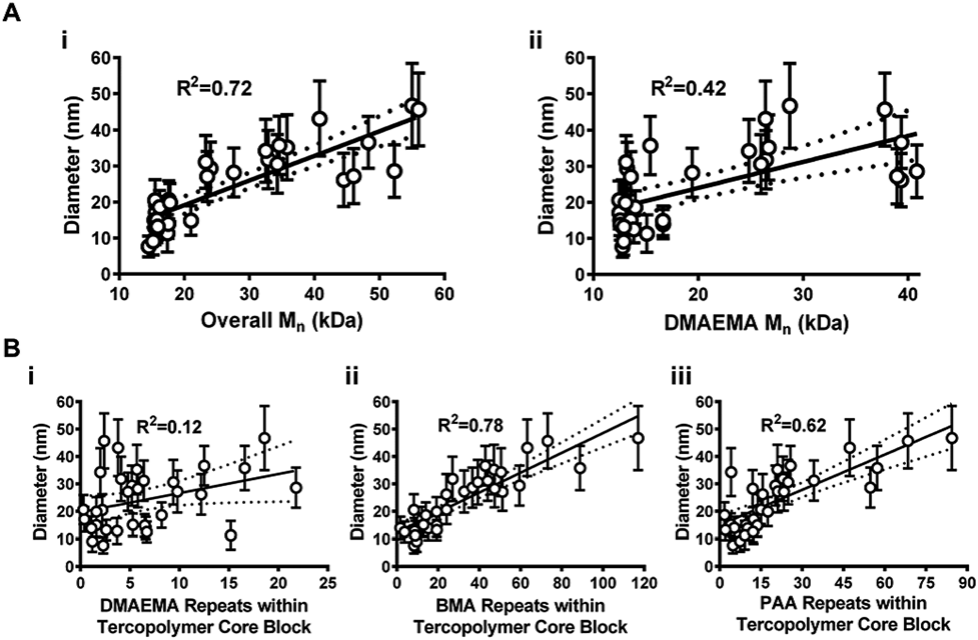
NP size as a function of copolymer *M*_n_ and monomer incorporation within hydrophobic cores. (A) Scatterplots of measured NP diameter *versus* (i) overall copolymer *M*_n_ and (ii) DMAEMA *M*_n_. (B) Scatterplots of NP diameter *versus* monomer repeats within NP hydrophobic cores for (i) DMAEMA, (ii) BMA, and (iii) PAA. Data shown as mean ± standard deviation from *n* = 3 independent measurements. Dotted lines indicate 95% confidence intervals.

To reduce special variation in the process, we assessed the synthetic reproducibility of key CQAs for one diblock copolymer as the following process variables were changed: personnel, analytical equipment (*e.g.*, GPC column), and reaction scale (*e.g.*, increased 10-fold). Stemming from previous work,^20,21^ NPs were synthesized with characteristics optimized for anti-biofilm drug delivery: Block 1 *M*_n_ of ∼12.5 kDa, corona-to-core molecular weight ratios of ∼4, and small diameters (<30 nm). As shown in Fig. 3, three separate researchers synthesized a total of eleven different diblock copolymer batches. Block 1 *M*_n_ and Block 2 *M*_n_ results were consistent across the batches, irrespective of researcher (Fig. 3A) or GPC analytical column (Fig. S4†), including the sixth batch synthesized by researcher A (*i.e.*, batch A6), which was scaled-up 10× as a pilot batch. The reaction and lyophilization yields (Fig. 3B) were similar for all batches, and no significant differences were found among diameters (Fig. 3C) or zeta potentials (Fig. 3D). Furthermore, the differences of ± 5 nm in NP diameter observed here were well within the previously observed NP size range (*i.e.*, 10–30 nm) that yielded identical antibacterial efficacy reduction of ∼2 log colony forming unit per mL using both planktonic and biofilm *in vitro* assays.^21^ Taken together, results demonstrated synthesis reproducibility for one NP design in terms of personnel, analytical equipment, and reaction scale while exemplifying RAFT polymerization durability across a range of conditions.

**Fig. 3:**
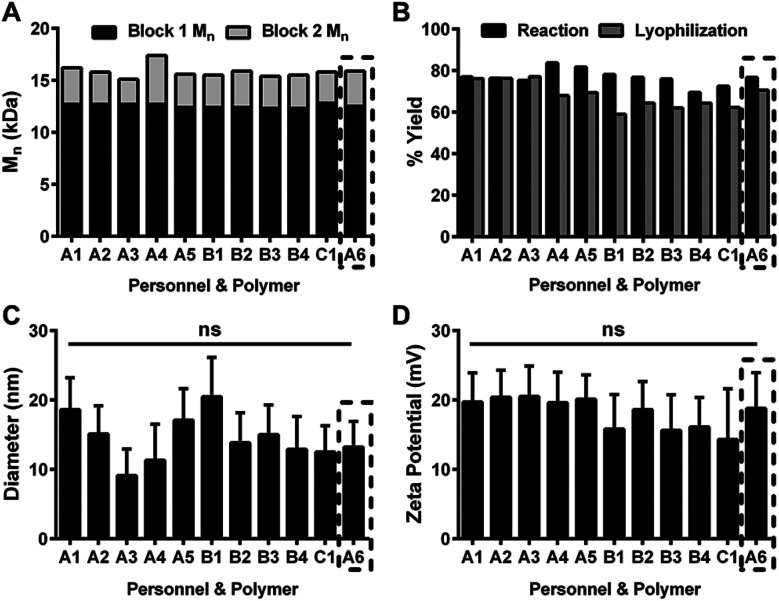
Reproducibility of diblock copolymer NP synthesis. (A) Block 1 and Block 2 *M*_n_ data for eleven NP batches synthesized by different personnel (*e.g.*, A, B and C). (B) Reaction and lyophilization yields for each NP batch. NP size (C) and zeta potential (D) data shown as the mean ± standard deviation from *n* = 3 (C) or *n* = 5 (D) measurements. ns = no significant difference from One-way ANOVA with Tukey's multiple comparisons test. Black dashed-line box indicates 10× scale-up pilot batch showing similar results to the other batches.

To further confirm NP synthesis robustness, an additional statistical process control (SPC) technique was pursued. Specifically, the process performance window was developed based on the data in Fig. 2A(i). Then, theoretical NP diameter values were estimated using a model that combined volumetric approximations based on the density and estimates of each monomer present within diblocks in Fig. 2A(i). The results of this predictive modeling strategy, shown in Fig. 4A, aligned well with the actual measurement data, encompassing the measured diameters for all but two polymers (*i.e.*, ∼94% probability of success). Therefore, the modeled mean average and standard deviation diameter values for the eleven batches shown in Fig. 3 (indicated by the dashed-line box in Fig. 4A) were used to assess the SPC of NP production using established methodology presented elsewhere.^38^ SPC evaluations require normally distributed data, which was the case for the eleven polymers included in Fig. 4A per Pearson's normality test (*p* = 0.84). Therefore, an initial set of synthesized batches (termed Alpha batches akin to Fig. 1) were evaluated for process performance in terms of NP diameter. In alignment with established SPC methodology,^38^ lower and upper specification limits (*i.e.*, LSL and USL) were set as the mean ± 3 standard deviations using the predictive model data for the Alpha batches. Batch sizes were then plotted and the process performance index (*P*_pk_), which is a measure of the initial process capability before corrective SPC actions are implemented, was calculated (Fig. 4B). The *P*_pk_ result of 0.99 indicated that the initial process was not at an adequate level of statistical control (*i.e.*, *P*_pk_ < 1.67).^38^ This result was corroborated by the level of variation observed in the Alpha batch results, including one batch (A3) where neither the mean nor standard deviation range overlapped with the process mean (*i.e.*, xbar). By applying insights obtained from the models presented in Fig. 1 and 2, clarity around key design parameters, such as Block 1 and Block 2 theoretical DP, improved control over the polymer synthesis process. These results were evident for the Beta batches synthesized chronologically after the Alpha group (Fig. 4B). In particular, the diblock copolymer NPs were within the specification limits. Moreover, the process capability index (*C*_pk_) value of 1.35 demonstrated that the process was under satisfactory statistical control (*i.e.*, *C*_pk_ ≥ 1.33),^38^ and the standard deviation range for every batch overlapped with the process mean. Importantly, *C*_pk_ is a statistical measure that corresponds to the capability of a process to produce outcomes within a desired specification range and can also provide an estimate of future process performance.^38^

**Fig. 4:**
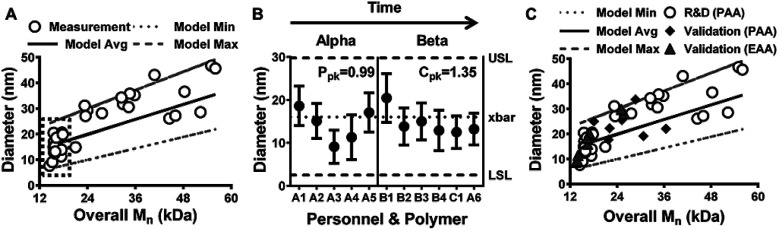
Predictive model and process capability analysis demonstrate statistical process control for NP production. (A) Model results show theoretical NP diameters, observed slope, and 95% confidence interval from Fig. 2A. White circles represent data means and the black, dashed box highlights batches from Fig. 3. (B) Process performance analysis of five Alpha batches and process capability analysis of seven Beta batches from Fig. 3 showing initial process performance (*P*_pk_ = 0.99) and the modified process capability (*C*_pk_ = 1.35) for diblock copolymer NP synthesis. Statistical process control (*P*_pk_ values ≥ 1.67 and *C*_pk_ values ≥ 1.33) was achieved only after application of insights from the model shown in Fig. 1. (C) Validation results comparing theoretical model data from (A), termed “R&D (PAA)” (white circles) with measurements for prospectively synthesized diblock copolymers containing either propyl acrylic acid (“Validation (PAA)”, black diamonds) or ethyl acrylic acid (“Validation (EAA)”, grey triangles) in the hydrophobic core. 6 out of 7 Validation (PAA) batches and all 8 Validation (EAA) batches were within model limits.

Once a process is under statistical control and shows only normal causes of variability, it becomes predictable and more relevant for clinical translation and commercialization.^11,38^ However, process performance qualification and validation are required before any product is approved for clinical use.^39^ As a simple validation approach, predictive model parameters (*e.g.*, model average, minimum, and maximum) from Fig. 4A were prospectively applied to seven additional *p*(DMAEMA)-*b-p*(DMAEMA-*co*-BMA-*co*-PAA) diblock copolymer NPs and eight diblock copolymer NPs designed and synthesized with PAA replaced by ethyl acrylic acid (EAA) in the hydrophobic core. As shown by black diamonds in Fig. 4C, 6 out of 7 (∼86%) PAA-containing NPs synthesized and characterized by different personnel, “E”, yielded size measurements within the predictive model size range. Additionally, size measurements for all 8 (∼100%) of the EAA-containing NPs synthesized and characterized by a combination of different personnel, “A” and “F”, were within the predictive model size range (grey triangles in Fig. 4C). These size measurements for the EAA NPs also aligned with the PAA NPs (Fig. 3 and the black dashed-line box in Fig. 4A), which had similar overall *M*_n_ values. These results validated the predictive model, thereby indicating model versatility and RAFT polymerization process robustness with respect to personnel and materials used. Therefore, this model coupled with the process capability analysis provided key process design insights and a robust predictive tool to facilitate future translation of polymer NP technology.

Additional continuous improvement efforts are needed to reach the full potential of this NP platform. For example, evaluation of residual data from the predictive model shown in Fig. 4A revealed a non-normal distribution across the *M*_n_ range tested (Fig. S5†). This result may be due to the small number of batches with *M*_n_ > 20 kDa. However, if needs arise for larger copolymers, additional batch synthesis and SPC analysis will be needed. Furthermore, other CQAs (*e.g.*, critical micelle concentration, corona-to-core molecular weight ratios, and/or p*K*_a_) or Block 2 monomer substitution could be evaluated and more extensive predictive models could be designed. As shown in Fig. S6,† exploration of these options has begun, but more work is needed to support additional modeling approaches.

Many existing tools and standardized techniques can be used to apply continuous process improvement and modeling practices. A list of several tools and techniques commonly used in LSS and QbD initiatives are shown in Table S1.† These tools can be used to define system wide processes (*e.g.*, process mapping, spaghetti diagrams), identify root causes of special cause variation or process deviations (*e.g.*, Ishikawa/Fishbone diagrams, 5 Why analysis), and manage risks (*e.g.*, Effort/Impact Matrix, FMEA). Additionally, implementation of simple tools, such as Poka Yoke (*i.e.*, mistake proofing), through the use of visual aids or other means can improve the reproducibility and efficiency of seemingly trivial, repetitive tasks (which are common in NP synthesis and characterization processes and can be overlooked during preclinical research efforts).^40,41^ For example, to minimize variability associated with NP dialysis purification processes, a simple piece of tape of a specific length can be adhered to a laboratory bench and used as a Poka Yoke to quickly and reproducibly measure tubing segments for dialysis (Fig. S7†). Given the stark influence that small details, such as tubing length, can have on routine dialysis procedures,^42,43^ the use of simple standardization tools, such as Poka Yoke, can greatly reduce confounding process variability and are commonly used in commercial and industrial settings.^40,41^ Extending the use of these tools to more research settings, particularly for nanotechnology research, may facilitate clinical translation and commercialization of nanomedicine research.

In summary, an established NP platform was used to successfully demonstrate how standardized systems level continuous improvement methodology can enhance the rigor and reproducibility of polymer NP research. Models linked key design parameters, such as theoretical Degree of Polymerization, to NP CQAs, such as polymer *M*_n_ and NP diameter, ultimately improving SPC of NP production from a sub-par *P*_pk_ value (<1.67) to a satisfactory *C*_pk_ value (≥1.33). Use of these and similar tools and techniques will foster more rigorous experimental practices and help achieve more reproducible results to facilitate translation of nanomedicines into clinical practice and/or industrial commercialization.

## Conflicts of interest

There are no conflicts to declare.

## Supplementary Material

RA-010-C9RA10091A-s001
