# Supplementary material for: Rigor and reproducibility in polymer nanoparticle synthesis and characterization
Source: RSC Adv. 2020 Jan 14;10(5):2513–8. doi: 10.1039/c9ra10091a (PMC8496373; doi:10.1039/c9ra10091a)
Supplement: RA-010-C9RA10091A-s001 [file RA-010-C9RA10091A-s001.pdf]

Electronic Supplementary Material (ESI) for RSC Advances.
This journal is © The Royal Society of Chemistry 2020

Supplementary Information

Title: Rigor and Reproducibility in Polymer Nanoparticle Synthesis and Characterization

Authors: *Kenneth R. Sims Jr., Julian P. Maceren, Alexander Ian Strand, Brian He, Clyde Overby, and Danielle S. W. Benoit.**

Materials

All materials were supplied by Sigma-Aldrich unless otherwise specified. Dimethylaminoethyl methacrylate (DMAEMA) and butyl methacrylate (BMA) were purified by distillation prior to use. Azobisisobutyronitrile (AIBN) was recrystallized from methanol. The chain transfer agent (CTA) used for reversible addition–fragmentation chain transfer (RAFT) polymerization, 4-cyano-4-[(ethylsulfanylthiocarbonyl)sulfanyl]pentanoic acid (ECT), ethyl acrylic acid (EAA), and propylacrylic acid (PAA) were synthesized as described previously [1-4]. All water used was deionized and distilled with resistivity of 18 MΩ, and all PBS used was 1× DPBS.

Polymer Synthesis, Purification, and Storage

The first block (*i.e.*, Block 1) synthesis consisted of combining dimethylaminoethyl methacrylate (DMAEMA) with 4-cyano-4-[(ethylsulfanylthiocarbonyl)sulfanyl]pentanoic acid (ECT) as the chain transfer agent (CTA) and 2,2-azobisisobutyronitrile (AIBN) as the initiator in dimethylformamide (DMF). The reaction vessel was purged with nitrogen for 45 minutes and subsequently reacted at 60°C for 6 hours. The product was precipitated 4 times in 80:20 pentane:diethyl ether using centrifugation and dried *in vacuo*. After characterizing Block 1 using gel permeation chromatography and proton nuclear magnetic resonance, the Block 1 p(DMAEMA) served as the macroCTA and AIBN served as the initiator for synthesis of Block 2, which consisted of DMAEMA, butyl methacrylate (BMA), and propyl acrylic acid (PAA). The Block 2 reaction reacted at 60 °C for 24 hours after purging the vessel with nitrogen for 45 minutes. Using centrifugation, the product was precipitated 4 times in 80:20 pentane:diethyl ether and then dried *in vacuo*. A CTA to initiator ratio of 5 was used for all polymers in this study.

Dried diblock copolymer was dispersed in ~5 mL 100% ethanol and diluted with ~25 mL PBS. The solution was transferred into pre-wetted 6-8 kDa dialysis membrane tubing measured to be six inches long before clipping each end. Dialysis occurred for ≥ 4 days with multiple water changes per day (8-10 changes total) to remove contaminants. The purified solution was frozen at -80 °C and lyophilized for ≥ 4 days using a Labconco FreeZone 2.5 freeze dryer. The lyophilized polymer was stored in closed 50 mL conical tubes at room temperature until use.

Polymer Characterization

Molecular weights of first block and diblock copolymers were determined by gel permeation chromatography (GPC, Shimadzu Technologies) using a miniDAWN TREOS multi-angle light scattering detector (Wyatt Technology) in line with an Optilab T-rEX refractive index detector (Wyatt Technology). The mobile phase consisted of High Performance Liquid Chromatography (HPLC) grade DMF + 0.05 mM LiCl (0.2 μm filtered) with a flow rate of 0.35

mL min^{-1} through a TSKgel SuperH-H guard column and TSKgel SuperHM-N column (Tosoh Biosciences) at 60 °C.

Nanoparticle Characterization

Nanoparticle size and zeta potential were measured using a Zetasizer Nano ZS (Malvern Panalytical). Size measurements were performed via dynamic light scattering (DLS) analysis using lyophilized polymer concentrations of ~0.2–0.3 mg mL^{-1} fully dispersed in PBS and passed through a 0.45 μm PVDF aqueous syringe filter into disposable cuvettes. Zeta potential measurements were performed using polymer concentrations of ~0.2–0.5 mg mL^{-1} in 90:10 water:PBS solutions and filtered using 0.45 μm PVDF aqueous syringe filters into disposable p1070 capillary cells.

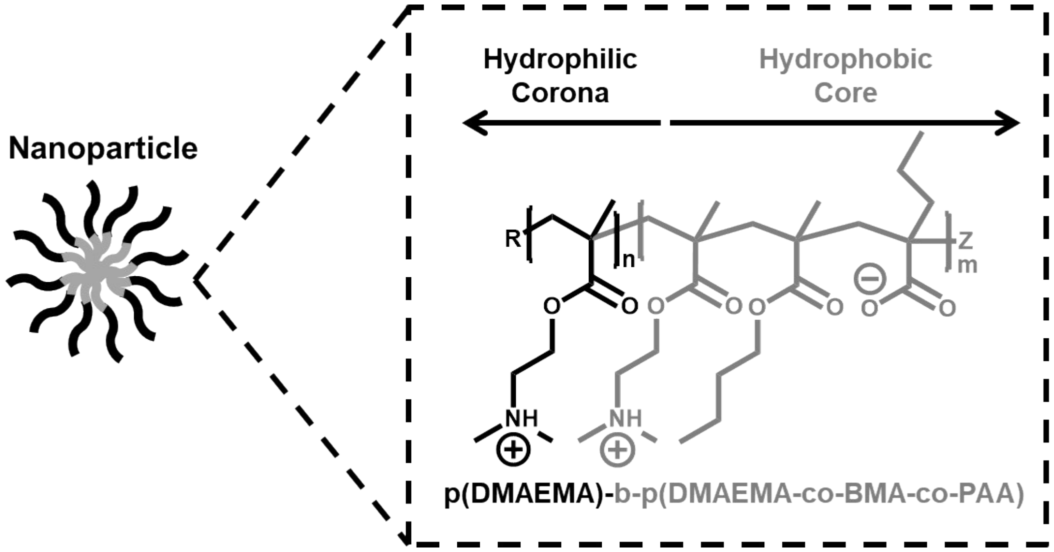

Figure S1. **Diblock copolymer nanoparticle platform used in this study.** Cartoon showing diblock copolymer nanoparticle with hydrophilic corona (black) and hydrophobic core (grey). Boxed contents show the polymer structure and components. Abbreviations: DMAEMA, dimethylaminoethyl methacrylate; BMA, butyl methacrylate; PAA, propyl acrylic acid.

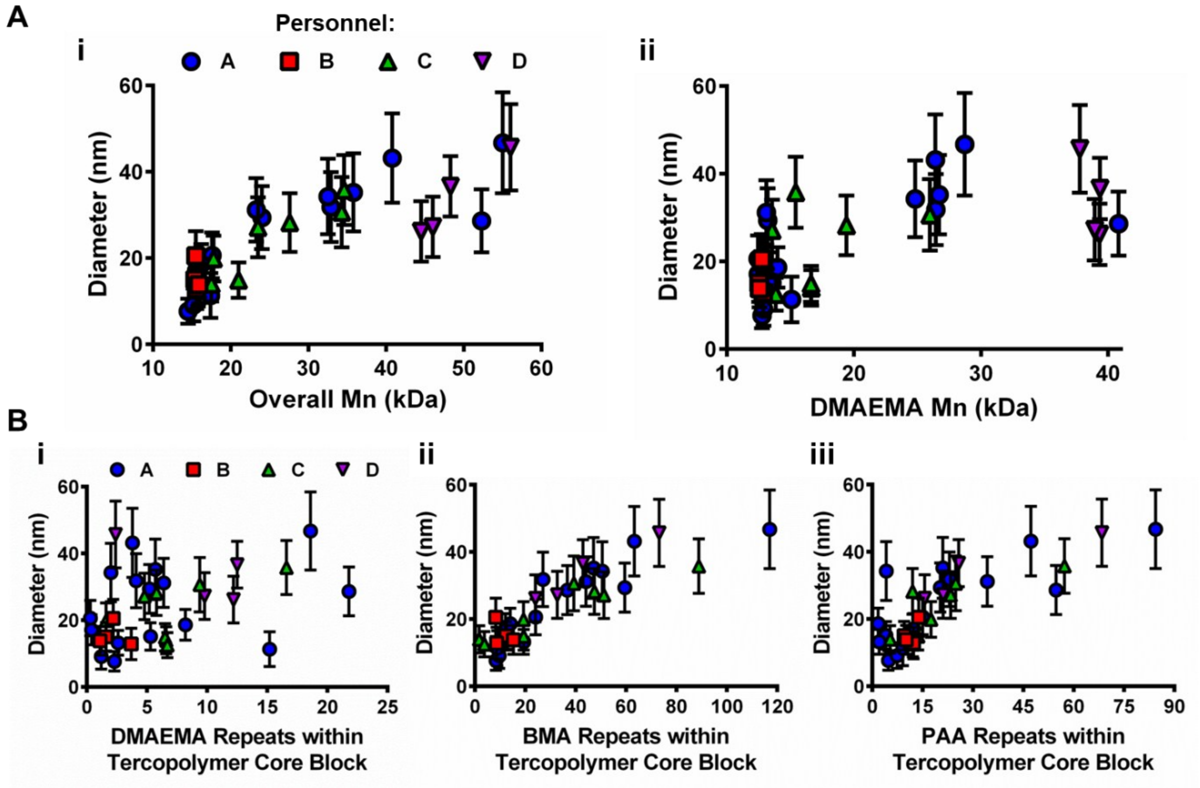

Figure S2. Nanoparticle size characterization as a function of copolymer molecular weight and monomer prevalence within the hydrophobic core stratified by personnel. A) Scatterplots showing measured nanoparticle diameter as a function of (i) overall copolymer M_n and (ii) DMAEMA M_n and stratified by personnel. B) Scatterplots of nanoparticle diameter versus monomer repeats in the nanoparticle hydrophobic core for (i) DMAEMA, (ii) BMA, and (iii) PAA stratified by personnel (*e.g.*, A, B, C, D). Data shown as mean ± standard deviation from n = 3 independent measurements for size.

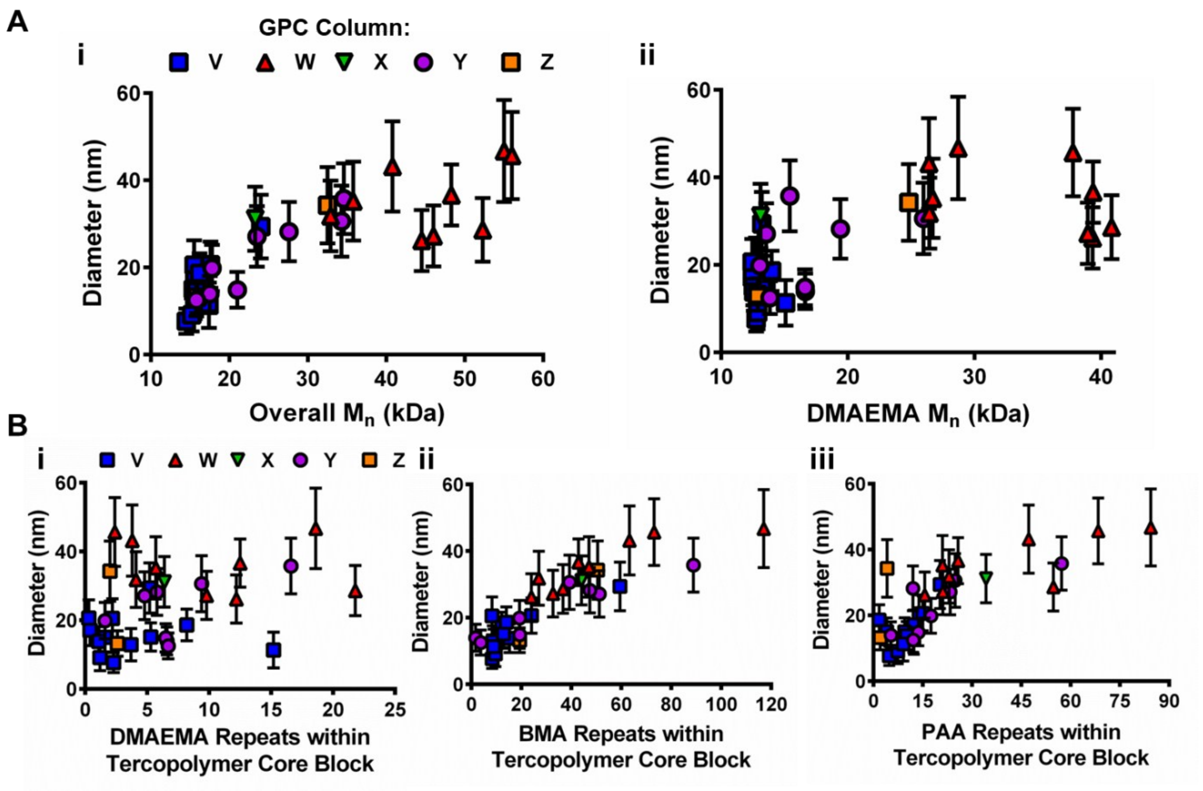

Figure S3. Nanoparticle size characterization as a function of copolymer molecular weight and monomer prevalence within the hydrophobic core stratified by GPC analytical column. A) Scatterplots showing measured nanoparticle diameter as a function of (i) overall copolymer M_n and (ii) DMAEMA M_n and stratified by GPC analytical column used for molecular weight determination. B) Scatterplots of nanoparticle diameter versus monomer repeats in the nanoparticle hydrophobic core for (i) DMAEMA, (ii) BMA, and (iii) PAA stratified by GPC analytical column (*e.g.*, V, W, X, Y, Z). Data shown as mean ± standard deviation from n = 3 independent measurements for size.

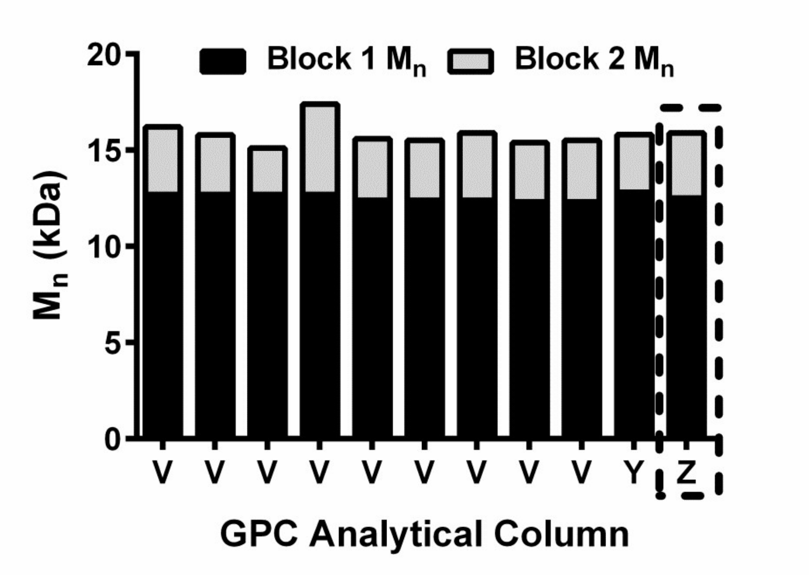

Figure S4. Reproducibility of diblock copolymer nanoparticle production for a single nanoparticle design stratified by GPC analytical column. A) Block 1 M_n and Block 2 M_n data for eleven batches of nanoparticles analyzed using three different GPC analytical columns (*e.g.*, V, Y, Z). These nanoparticles were designed to have Block 1 M_n of ~12.5 kDa, corona-to-core molecular weight ratios (CCRs) of ~4, and small diameters (< 30 nm). Black dashed-line box indicates a 10x scale-up (from hundreds of milligrams to grams of product) pilot batch showing similar characteristics to the other batches.

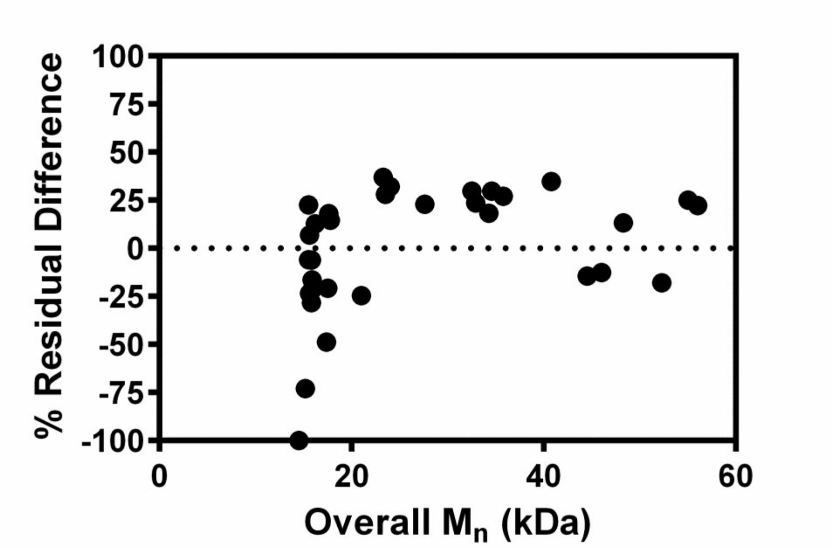

Figure S5. Percent residual difference analysis highlights degree of data normality. A residual difference plot showing the percent difference between measured and predicted nanoparticle diameters. The results show normally distributed size data for polymer nanoparticles with overall M_n < 20 kDa and non-normal data distribution for M_n > 20 kDa. Production of additional batches with M_n > 20 kDa will be necessary to evaluate statistical process control for nanoparticles with these molecular weight characteristics.

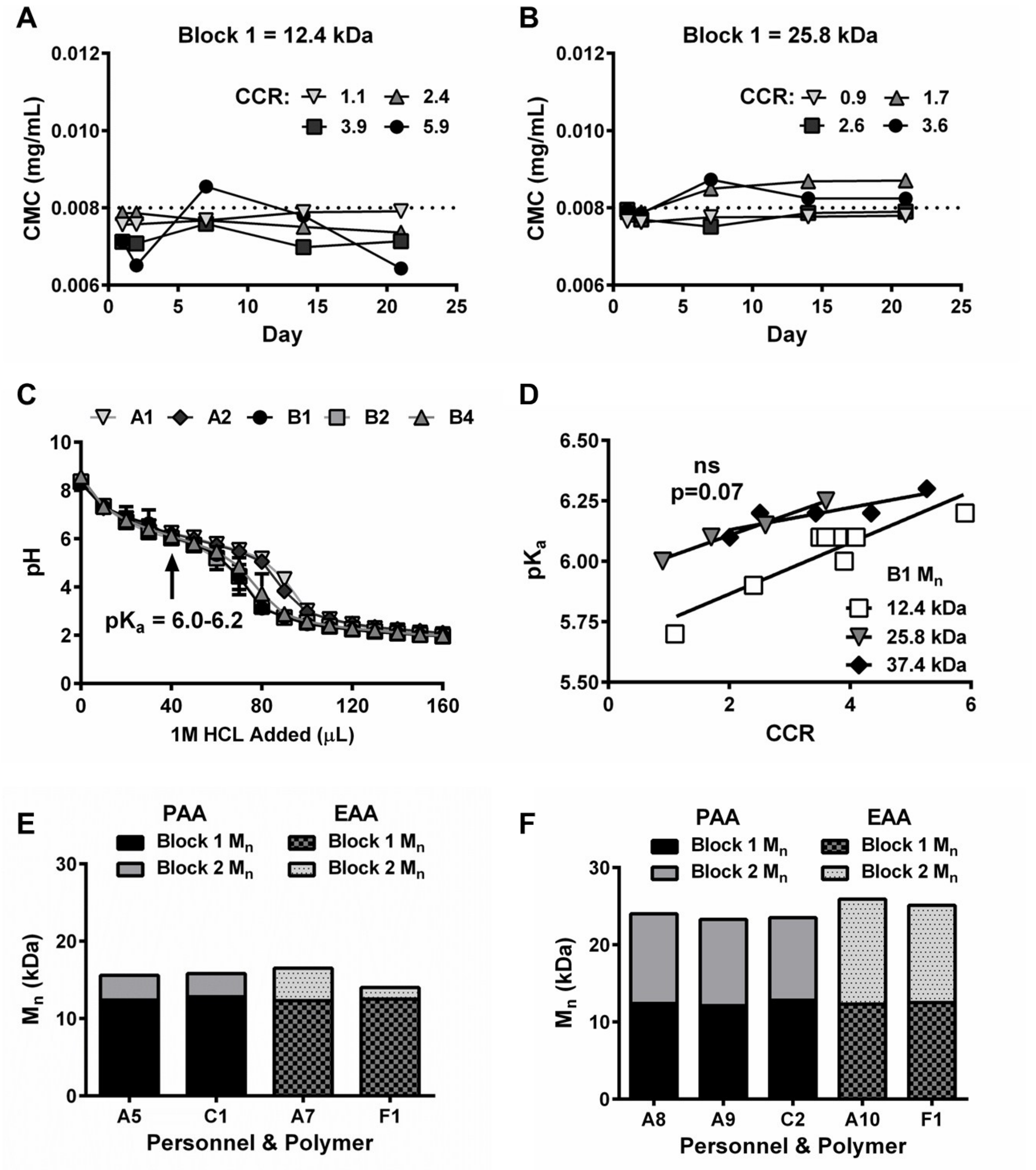

Figure S6. Multiple product attributes offer opportunities for predictive modeling to enhance rigor and reproducibility of nanoparticle designs. A) Critical micelle concentration (CMC) test results over 21 days for diblock copolymers with Block 1 M_n = 12.4 kDa and varying corona-to-core molecular weight ratios (CCRs). B) CMC test results over 21 days for diblock copolymers with Block 1 M_n = 25.8 kDa and varying CCRs. C) pH titration curve for multiple batches from Figure 3 highlighting the reproducibility of the demonstrated pK_a values. D) Scatterplot of pK_a vs. CCR stratified by Block 1 Mn showing no significant difference among the slopes of the lines. R^2 values of 0.84, 0.85, and 0.75 were obtained for Block 1 M_n 12.4 kDa, 25.8 kDa, and 37.4 kDa, respectively. E) Block 1 M_n and Block 2 M_n data for four batches of nanoparticles synthesized by different personnel (*e.g.*, A, C, F) stratified by monomer differences in the hydrophobic core. Polymers A5 and C1 contained PAA in their cores, and Polymers A7 and F1 contained EAA. These nanoparticles were designed to have Block 1 M_n of ~12.5 kDa and CCRs of ~4 as in Figure 3. These results indicate the nanoparticle production process reproducibility extends to polymers containing monomer substitutions in Block 2. F) Block 1 M_n and Block 2 M_n data for four batches of nanoparticles synthesized by different personnel (*e.g.*, A, C, F)

stratified by monomer differences in the hydrophobic core. Polymers A5 and C1 contained PAA in their cores, and Polymers A7 and F1 contained EAA. These nanoparticles were designed to have Block 1 M_n of ~12.5 kDa and CCRs of ~1. These results indicate the nanoparticle production process reproducibility not only extends to polymers containing monomer substitutions in Block 2 but is also independent of nanoparticle CCR.

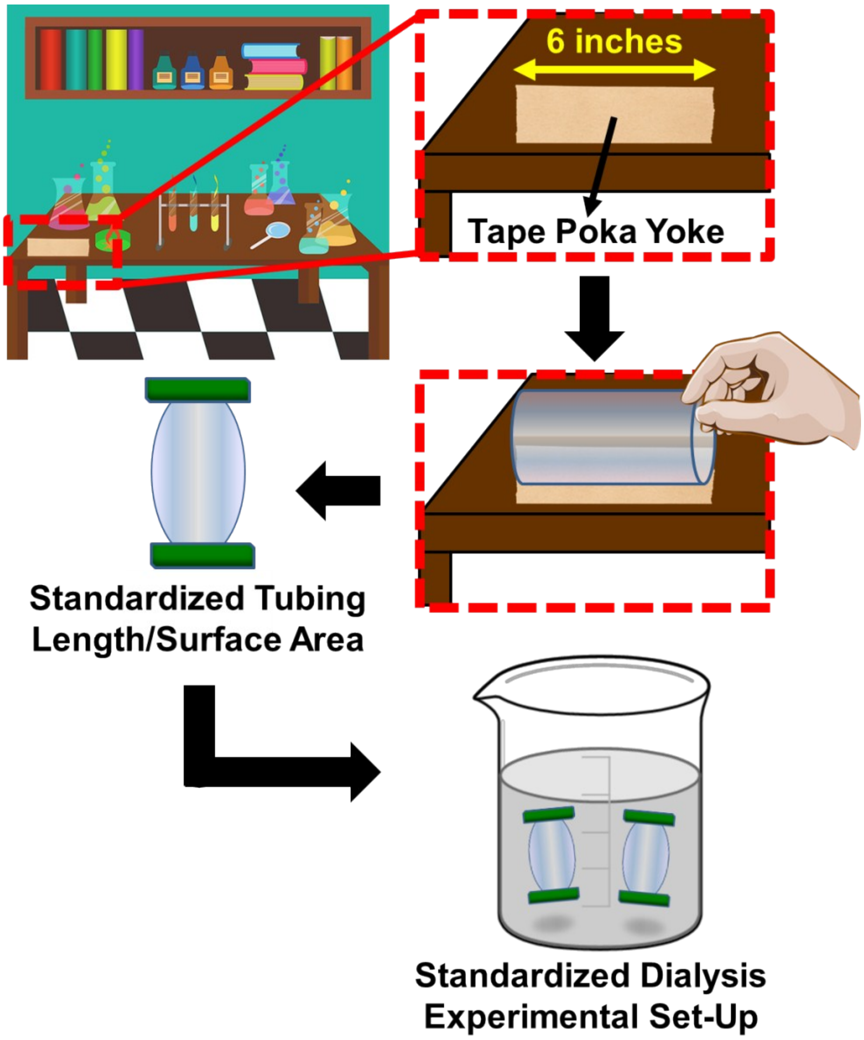

Figure S7. Cartoon example of a practical Poka Yoke laboratory application. The concept of Poka Yoke is shown through a simple example where a piece of tape of a specific length (*e.g.*, 6 inches) is adhered to a surface (*e.g.*, lab bench) and used to quickly and reproducibly measure tubing segments for dialysis experiments. By standardizing the length and therefore, the surface area for dialysis experiments, confounding process variability known to be inherent to dialysis procedures [5-6] can be controlled or significantly reduced.

Table S1. Commonly used process improvement tools and techniques

Tool/Technique	Description	Purpose
**SIPOC**	A high level process map to understand relationships among people, materials, process steps, and products.	Defines Suppliers, Inputs, Process, Outputs, and Customers
**Spaghetti Diagram**	A visual map that traces resource movement or activity in a process.	To help identify redundant actions and drive efficiencies in a process work flow.
**Ishikawa Diagram**<br><br>**(Fishbone Diagram)**	A brainstorming tool structured as a type of cause and effect diagram.	To help visualize and systematically sort through all possible causes of a problem.<br><br>Multiple standard categories generally address most problems:<br><br>6Ms<br>• Machines<br>• Man<br>• Materials<br>• Measurements<br>• Methods<br>• Mother Nature<br><br>8Ps<br>• People<br>• Place<br>• Policies<br>• Price<br>• Process<br>• Procedures<br>• Product<br>• Promotion
**5 Why Analysis**	A common investigative tool to drill down to the next level cause(s) of a problem by asking "Why?" five times.	To ideally isolate the sole root cause and identify any contribution factors of a problem.
**Kaizen**	A common continuous improvement technique to repeatedly focus efforts and attack small problems	To rapidly achieve process improvements by identifying and eliminating waste,

Tool/Technique	Description	Purpose
	in a process.	procedural gaps, and sources of process variation.
**Poka Yoke**	A mistake proofing tool that leverages simple visual controls or other mechanisms.	To prevent mistakes and reduce sources of variation as well as facilitate discovery of procedural errors or process deviations.
**Effort/Impact Matrix**	A common project management and decision making tool that filters out solutions to a problem which might not be worth the effort to complete.	To drive decision making regarding action prioritization.
**FMEA**	Failure Modes and Effects Analysis is a common process improvement and risk management tool.	To identify areas where a process or system can fail, estimate the risks associated with failure, identify and prioritize actions to reduce the risks, and evaluate control mechanisms.
**Process Capability Analysis**	A common process improvement tool used for statistical process control.	To measure the ability of a process to produce an outcome within desired specifications.

Conflicts of interest

There are no conflicts to declare.

Acknowledgements

The authors gratefully acknowledge the National Institutes of Health (R01 DE018023 to DB and F31 DE026944 to KS) and the National Science Foundation (DMR 1206219 to DB) for funding that supported this work. Research reported in this publication was supported by the National Institute of Dental & Craniofacial Research of the National Institutes of Health. The content is solely the responsibility of the authors and does not necessarily represent the official views of the National Institutes of Health. The authors would also like to thank James L. McGrath for access to the Malvern Zetasizer and Sidney Duquette for his assistance with several polymer syntheses and providing feedback on initial versions of the polymer synthesis SOPs used for this work.

Supporting Information References

[1] A. J. Convertine, D. S. Benoit, C. L. Duvall, A. S. Hoffman, P. S. Stayton, *Journal of Controlled Release*. **2009**, 133, 221.

[2] G. Moad, Y. K. Chong, A. Postma, E. Rizzardo, S. H. Thang, *Polymer*. **2005**, 46, 8458.

[3] N. Murthy, J. R. Robichaud, D. A. Tirrell, P. S. Stayton, A. S. Hoffman, *Journal of Controlled Release*. **1999**, 61, 137.

[4] M. S. a. T. Ferritto, D. A., in *Macromolecular Syntheses*, Vol. 11, Wiley, New York, NY **1992**, 59.

[5] S. A. Abouelmagd, B. Sun, A. C. Chang, Y. J. Ku and Y. Yeo, *Molecular pharmaceutics*, **2015**, 12, 997-1003.

[6] S. Modi and B. D. Anderson, *Molecular pharmaceutics*, **2013**, 10, 3076-3089.
